# From Fundamental Self-Assembly Studies to Applications in Everyday Life: The Formation of a Supramolecular Shampoo

**DOI:** 10.3390/gels12070589

**Published:** 2026-07-02

**Authors:** Sofia Chinelli, Roberta Stile, Demetra Giuri, Claudia Tomasini

**Affiliations:** Dipartimento di Chimica Giacomo Ciamician, Università di Bologna, Via Piero Gobetti, 85-40129 Bologna, Italy; sofia.chinelli2@unibo.it (S.C.); roberta.stile2@unibo.it (R.S.)

**Keywords:** supramolecular gels, self-assembly, lipoamino acid, surfactants, rheology

## Abstract

Amino acid-based surfactants are promising ingredients for cosmetic formulations, combining mildness with intrinsic self-assembly properties. A recent challenge in the cosmetic field is the replacement of synthetic polymers, used as rheological modifiers, with sustainable and biodegradable alternatives. In this work, sodium cocoyl glycinate (SCG) and sodium cocoyl alaninate (SCA) were investigated as both surfactants and supramolecular gelators for the development of a “supramolecular shampoo”. pK_a_ analysis and rheological studies revealed that SCG forms robust gel networks at pH 5, whereas SCA shows limited stability. The progressive incorporation of typical cosmetic ingredients, including cocamidopropyl betaine (CAPB), preservatives, conditioning agents, and fragrance, led to a controlled decrease in mechanical strength while preserving pseudoplastic behavior. The final formulation remained stable under accelerated aging and freeze–thaw conditions for months. These results demonstrate that supramolecular structuring offers a viable and sustainable alternative to conventional polymer-based systems in shampoo formulations.

## 1. Introduction

The earliest cleansing surfactants were soaps, obtained by saponifying natural fats and used since ancient times [1]. Despite progressive refinements, such as the introduction of vegetable oils, traditional soaps suffered from intrinsic limitations, including high alkalinity and poor performance in hard water [2]. The development of synthetic detergents in the late 1950s, followed in the 1990s by liquid systems based on SLES/CAPB (SLES: sodium laureth sulfate; CAPB: cocamidopropyl betaine), revolutionized personal cleansing due to their strong foaming properties, stability, and cost-effectiveness [3,4]. However, growing concerns over sulfates and increasing demand for milder and more sustainable formulations have accelerated the search for alternative surfactant systems [4,5,6,7,8,9]. At the same time, rheological control has become a crucial parameter in shampoo formulations, directly influencing stability, application behavior, and consumer perception. Surfactant solutions evolve from Newtonian behavior to shear-thinning and viscoelastic regimes at higher concentrations, due to micellar growth and entanglement [10]. Ideal shampoos display pseudoplastic behavior (thick at rest, yet easily spread during use), a profile traditionally achieved through synthetic polyacrylates. Increasing environmental scrutiny and microplastic regulations now motivate the development of more sustainable structuring strategies [11], ideally based on reversible supramolecular organization rather than permanent polymeric networks [12,13].

In this context, amino acid-based surfactants, including glycinates, glutamates, sarcosinates, and taurates, have emerged as promising candidates owing to their mildness, biodegradability, and renewable origin [14,15,16]. Structurally obtained by acylation of amino acids, these amphiphiles retain essential molecular features of peptide systems, such as amide bonds, defined backbone geometry, and hydrogen-bonding capability [17,18]. The combination of a hydrophobic alkyl chain with an amino acid polar headgroup confers not only surface activity but also an intrinsic propensity for supramolecular self-assembly [19,20,21]. Thus, beyond their established role as cleansing agents, amino acid–based surfactants can be viewed as single-residue peptide mimics, capable of organizing into higher-order architectures through weak and reversible interactions [22]. Despite their widespread use, however, the extent to which such assemblies can generate formulation-relevant structuring in realistic shampoo matrices remains insufficiently understood.

The conceptual framework enabling this perspective is deeply rooted in the fundamental advances of peptide supramolecular chemistry [23,24,25,26,27,28]. Peptides represent uniquely programmable molecular systems: through rational sequence design, subtle variations in residue type and backbone constraint translate into controlled secondary structure formation and hierarchical assembly [29,30,31,32,33,34].

**Figure 1:**
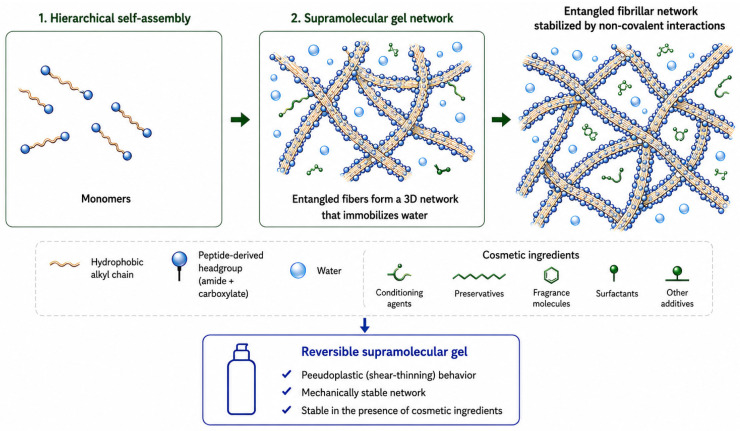
Schematic illustration of the proposed supramolecular assembly of SCG/SCA in supramolecular shampoo gels.

Building on these principles, the present study explores whether amino acids derived from amphiphiles can extend this design paradigm to complex, real-world formulations. Sodium cocoyl glycinate (SCG) and sodium cocoyl alaninate (SCA) are investigated in both model and realistic shampoo systems to evaluate their dual function as mild surfactants and supramolecular structuring agents [18,35,36], linking aggregation state to rheological performance. Here, “supramolecular shampoo” refers to a formulation in which reversible non-covalent interactions drive mesoscale organization (e.g., elongated/entangled aggregates) that translates into macroscopic pseudoplastic behavior. In doing so, this work aims to demonstrate how concepts originally developed within fundamental peptide chemistry can inform sustainable material design in everyday applications, bridging supramolecular theory and practical formulation science. Figure 1 shows a schematic cartoon of the proposed supramolecular assembly. The figure is intended to provide a concise visual overview of the system and to clearly summarize the main purpose and scope of the work.

## 2. Results and Discussion

In a recent study, we reported the preparation and characterization of simple surfactant-based cosmetic formulations at pH 5, the physiological pH of skin and scalp [22]. To make gels in the wide class of surfactants, including sulphonates and sulphates, we added Boc-L-Dopa(Bn)_2_-OH in 1% *w*/*v*, which we previously studied as a robust low molecular weight gelator under several conditions [32,37,38]. In most cases, the gelator self-assembled into fibers, inducing gel-like behavior confirmed by rheological analysis (G′ > G″). In two cases the gelling ability of two *N*-substituted amino acid surfactants, SCG and SCA, was confirmed at pH 5 and 10% *w*/*w* concentration even in the absence of our gelator. The 10% *w*/*w* concentration was selected considering that the total amount of surfactants in commercial shampoos usually does not exceed 20% [5]. In addition, since each commercial surfactant has a specific concentration range, different amounts were withdrawn to achieve 10% of active matter in the solution (see Table S1).

**Figure 2:**
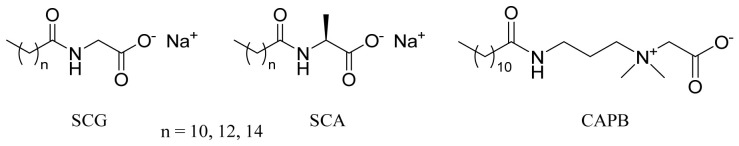
Chemical structure of SCG (sodium cocoyl glycinate), SCA (sodium cocoyl alaninate) and of cocamidopropyl betaine (CAPB).

We now want to demonstrate that commercial molecules such as SCG and SCA can behave as gelators under some given conditions, thus allowing the formation of a “supramolecular shampoo”. The great advantage of these materials is that they are cheap ingredients, already produced on a large scale, and normally used by cosmetic companies. These molecules are mild amino acid–based anionic surfactants derived from renewable feedstocks, combining fatty acids of coconut origin with naturally occurring amino acids (alanine and glycine, respectively). The term “cocoyl” refers to the acyl group obtained from coconut oil fatty acids—primarily lauric, myristic, and palmitic acids—which provide the hydrophobic tail of the molecule responsible for surface activity and self-assembly (Figure 2).

In our preliminary work, we noticed that surfactants derived from sulphonates and sulphates show no gelation ability. This different behavior can be attributed to variations in the pK_a_ values of the materials, a key parameter in predicting their self-assembly properties [33]. Sulphonates typically exhibit pK_a_ values around −5, whereas carboxylates generally display pK_a_ values of ~4 or higher. Notably, the pK_a_ of such compounds may vary with concentration and aggregation state, as commonly observed for amphiphilic systems, leading to values that can differ significantly from those of small carboxylic acids such as acetic acid [39,40]. To determine the experimental pK_a_ values of SCG and SCA, both bearing a carboxylate moiety, their basic solutions were titrated with hydrochloric acid (HCl) [33,38].

**Figure 3:**
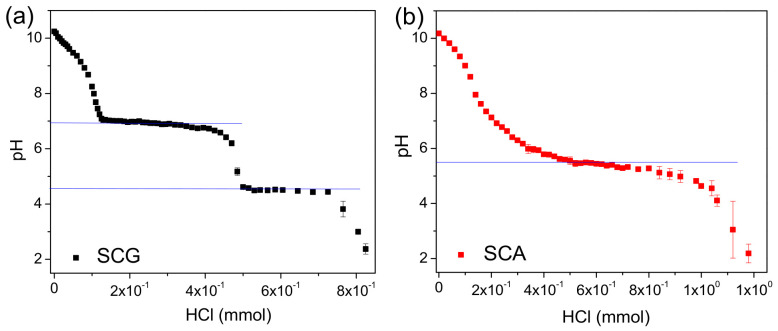
Titration curves (pH versus mmol of added HCl 1.0 M) of SCG ((**a**), black) and SCA ((**b**), red). The experiments were repeated in triplicate, and the results are expressed as mean ± standard deviation.

Figure 3 shows the results of titrating SCG and SCA at a 10.0% *w*/*w* concentration, that is the same concentration used in this work. We started at pH 10.2 and added small aliquots of 1 M HCl until the pH reached 3. Each experiment was performed in triplicate to ensure accuracy and reproducibility. The pK_a_ was defined as the pH at which 50% of the molecules are protonated. In most cases, this value appeared as a plateau on the pH titration curve.

The inspection of the titration profiles indicates that both molecules exhibit apparent pK_a_ values higher than the theoretical value of ~3.9, typical of small carboxylic acids that do not undergo self-assembly [40,41]. SCG displays two plateaus at pH 6.9 and 4.5. The former is significantly higher than the expected value, whereas the latter is in closer agreement. This behavior suggests that, upon acid addition, a reorganization of the surfactant micelles occurs, resulting in a different exposure and subsequent protonation of the carboxyl groups. In contrast, SCA exhibits a single plateau at pH 5.5, slightly higher than the theoretical value.

These observations imply that SCG and SCA may behave differently at pH 5.5, a value relevant for cosmetic formulations. At this pH, SCG is partially protonated and can form supramolecular fibers. Conversely, SCA has a pK_a_ close to this value, leading to competing equilibria that may hinder the formation of stable fibrillar structures required for supramolecular gelation.

Overall, while both surfactants are expected to form similar gels under more acidic conditions (pH 3–4), their behavior is likely to differ at pH ≥ 5. To verify this hypothesis, their gelation ability was investigated under conditions relevant to the formation of supramolecular “shampoo” systems.

In our previous paper [22], we reported the formation of gels from SCG and SCA at 10% *w*/*w*, with the final pH adjusted to approximately 5.0 using lactic acid. Rheological characterization, including amplitude and frequency sweep measurements, revealed that the SCG-derived gel exhibited a higher storage modulus than the SCA-derived counterpart (53.1 KPa vs. 13.4 KPa) (Table 1), indicating superior mechanical strength. This difference is consistent with the higher pK_a_ of SCG relative to SCA and its influence on gel properties. Notably, the pH of the system lies below the pK_a_ values of both SCG and SCA, suggesting that a similar molecular self-assembly mechanism governs gel formation in both cases. In this context, gelation is likely driven by hydrogen bonding, in conjunction with hydrophobic interactions among the long alkyl chains, leading to the formation of extended fibrillar networks.

To develop a supramolecular shampoo with a complete formulation, additional components were incorporated and the stability of the resulting gels was evaluated. Cocamidopropyl betaine (CAPB, Figure 2), a zwitterionic surfactant widely used in cosmetic formulations to improve softness and sensory properties, was selected as the first additive. The presence of both a positive charge and a negative charge in the chemical structure of CAPB can influence the supramolecular organization of the gel through electrostatic interactions and hydrophobic associations with the amphiphilic components of the network. These interactions may modify the packing of the self-assembled structures, potentially leading to a softer and more homogeneous material.

**Figure 4:**
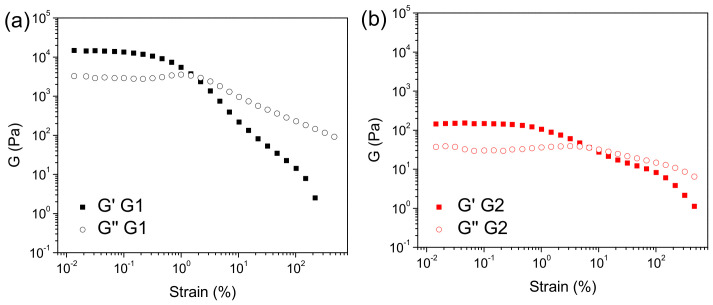
(**a**) Amplitude sweep of G1 (SCG 10% *w*/*w* and CAPB 4.5% *w*/*w*); (**b**) Amplitude sweep of G2 (SCA 10% *w*/*w* and CAPB 4.5% *w*/*w*).

Based on these considerations, two gels were prepared containing either SCG or SCA at a concentration of 10% *w*/*w*, with CAPB added at 4.5% *w*/*w*, a concentration commonly used in commercial shampoo formulations, and lactic acid to trigger gel formation. The rheological properties of these gels were first investigated to assess the effect of CAPB on the mechanical behavior of the systems, through amplitude sweep (Figure 4), frequency sweep and viscosity curves (Figure S1).

When SCG (10% *w*/*w*) was combined with the amphoteric surfactant CAPB (4.5% *w*/*w*), gel G1 was formed, as confirmed by amplitude sweep and frequency sweep measurements indicating the establishment of a relatively strong network. Similarly, the combination of SCA (10% *w*/*w*) with CAPB (4.5% *w*/*w*) yielded gel G2. Comparison of the amplitude sweep data for G1 and G2 (Figure 3 and Table 1) shows that G2 is significantly weaker than G1. This observation is consistent with the behavior of the two pure gelators, suggesting that minor variations in composition markedly influence SCA-based gels, likely due to the proximity of its pK_a_ to the gel pH. In contrast, SCG, characterized by a substantially higher pK_a_, appears more robust and less sensitive to formulation changes. The viscosity curves of the two gels, G1 and G2, shown in Figure S1, indicate that in both cases the viscosity decreases with increasing shear rate, confirming their pseudoplastic (shear-thinning) behavior. This occurs because the internal structure of the gels progressively aligns in the direction of flow under shear, reducing intermolecular interactions and, consequently, the resistance to deformation.

**Figure 5:**
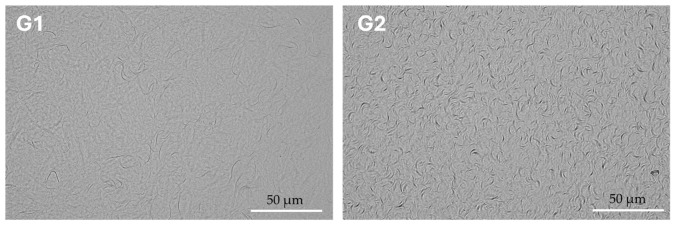
Optical microscope images of gels G1 (**left**) and G2 (**right**). Scalebar is 50 μm.

A deeper insight into the structure of the supramolecular assembly was obtained by the analysis of the wet gel with an optical microscope with a magnification of 40× (Figure 5). This technique was selected because it is rapid, straightforward, and provides reliable information without requiring gel lyophilization. Fiber formation is evident in both samples. G1 displays long, thin fibers, whereas G2 exhibits shorter, thicker fibers. These morphological differences may reflect differences in gel stability.

**Figure 6:**
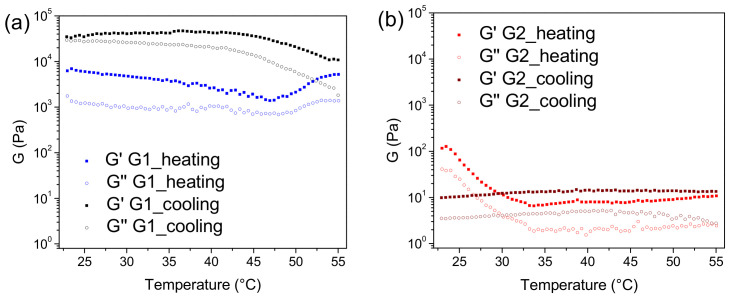
(**a**) Temperature sweep between 23 and 55 °C sweep of G1 (SCG 10% *w*/*w* and CAPB 4.5% *w*/*w*); (**b**) Temperature sweep between 23 and 55 °C sweep of G2 (SCA 10% *w*/*w* and CAPB 4.5% *w*/*w*).

A temperature sweep of G1 between 23 and 55 °C revealed a progressive weakening of the structure up to 46 °C (G′ decreasing from 6200 to 1400 Pa), followed by a subsequent increase, with the material nearly recovering its initial properties upon complete cooling (Figure 6). In contrast, the temperature sweep analysis of G2 further highlighted the thermal fragility of the system: the material behaved as a viscoelastic solid (G′ > G″) up to approximately 34 °C, after which a sharp decline in mechanical strength was observed (G′ decreasing from 116 to 6 Pa). Notably, the original mechanical properties were not recovered upon cooling.

**Figure 7:**
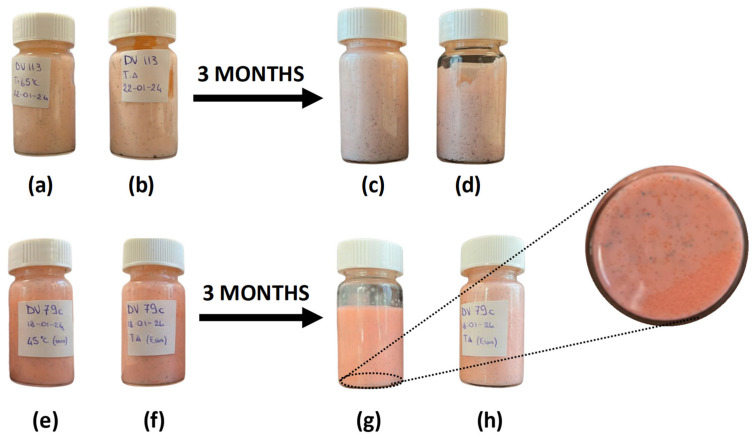
G1 (**top**) and G2 (**bottom**) following the addition of silica and jojoba particles to assess suspension capacity; a dye was included to enhance the visibility of the silica particles. (**Top**): G1 at T0 (immediately after preparation) at 45 °C (**a**) and room temperature (**b**), and after three months of storage at 45 °C (**c**) and room temperature (**d**). (**Bottom**): G2 at T0 (immediately after preparation) at room temperature (**e**) and 45 °C (**f**), and after three months of storage at 45 °C ((**g**); inset shows magnification of the vial bottom) and room temperature (**h**).

Additional insight was obtained from suspension tests performed at both room temperature and 45 °C. The suspending ability of the gels was evaluated using jojoba beads (density 0.85–0.91 g cm^−3^, less dense than water) and silica particles (density 1.6–1.8 g cm^−3^, denser than water) over a period exceeding three months. For gel G1 (Figure 7), both types of particles remained fully suspended under both conditions, demonstrating the excellent structuring capacity of the gel. These remarkable suspending properties are further evidenced by the retention of incorporated air, which remained trapped in the formulation as clearly visible bubbles, indicating the presence of a stable and well-developed network capable of supporting dispersed phases over extended periods.

In contrast, the gel G2 weakened significantly at 45 °C, as anticipated by the temperature sweep analyses, resulting in clear sedimentation of the heavier silica particles, while the lighter jojoba beads remained effectively trapped. Although G2 initially showed promising properties, it exhibited poor long-term stability during storage at 45 °C, with phase inhomogeneity appearing at the bottom of the sample after three months (Figure 5). Consequently, G1 was selected for further studies, as it ensured both robust gel formation and improved long-term stability.

After selecting the most stable system (SCG/CAPB, G1), additional components were incorporated to obtain a formulation more closely resembling a shampoo and to ensure stability in aqueous media. Among these, the inclusion of a preservative is essential to prevent microbial contamination and to guarantee product safety during storage and use.

**Figure 8:**
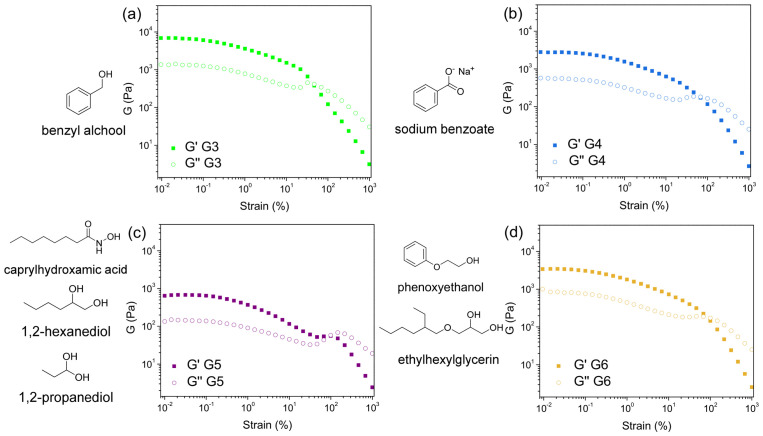
(**a**) Chemical structure of the preservative added for the formation of G3 and amplitude sweep analysis of G3; (**b**) chemical structure of the preservative added for the formation of G4 and amplitude sweep analysis of G4; (**c**) chemical structures of the preservatives added for the formation of G5 and amplitude sweep analysis of G5; (**d**) chemical structures of the preservatives added for the formation of G6 and amplitude sweep analysis of G6.

To identify a preservative system compatible with the supramolecular gel network, four formulations were prepared based on G1 by incorporating different preservatives [42] benzyl alcohol at 0.5% *w*/*w* (G3), sodium benzoate at 0.19% *w*/*w* (G4), a mixture of caprylhydroxamic acid/1,2-hexanediol/1,2-propanediol at 1.5% *w*/*w* (G5), and a mixture of phenoxyethanol/ethylhexylglycerin at 0.9% *w*/*w* (G6). The selected concentrations were chosen based on preservative efficacy and regulatory limits (see Section 4 for details) and were screened to assess their rheological compatibility, not their antimicrobial performance. The choice and amounts of these ingredients ensure compliance with Regulation (EC) No 1223/2009 on cosmetic products [43]. The mechanical properties of G3-G6 were evaluated by amplitude sweep (Figure 8), frequency sweep and viscosity analyses (Figures S2 and S3).

From the analysis of amplitude sweep, frequency sweep and viscosity measurements it can be concluded that, in all cases, the presence of preservatives does not hinder gel formation, although their impact on the rheological properties differs. Comparison of the storage modulus (G′) values reveals a moderate decrease for G3, G4, and G6 relative to G1 (Table 1), whereas a more pronounced reduction is observed for G5. This effect can likely be attributed to the higher preservative content in this gel. Among the systems investigated, G3 and G4 were selected for further studies, as they contain relatively low amounts of preservative (0.5% *w*/*w* and 0.19% *w*/*w*, respectively), minimizing their influence on the gel network.

**Figure 9:**
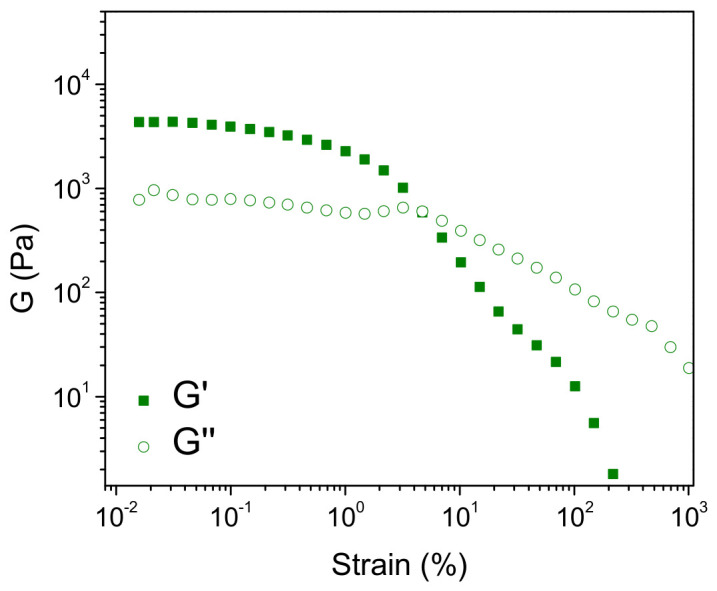
Amplitude sweep analyses of the gel G7, containing the two preservatives selected.

In addition, the combined use of benzyl alcohol and sodium benzoate was evaluated through the preparation of a new gel (G7) to assess any potential synergistic effects on gel stability. Amplitude sweep, frequency sweep and viscosity data (Figure 9 and Figure S4) indicate that G7 has a pseudoplastic behavior: its G′ remains on the order of 10^4^ Pa, and it is independent of frequency, confirming that the gel maintains satisfactory mechanical stability even in the presence of both preservatives.

**Figure 10:**
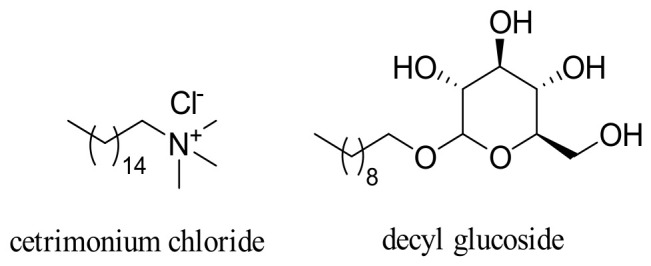
Chemical structure of the additional components of the shampoo.

Following these encouraging results, a supramolecular gel network was further developed by incorporating additional components commonly used in cosmetic formulations, with the aim of obtaining a system that more closely mimics a commercial shampoo. Specifically, cetrimonium chloride (a cationic conditioning agent included to improve spreadability) and decyl glucoside (a non-ionic surfactant) were introduced (Figure 10) as these ingredients are often present in shampoo to improve sensory and foaming properties (see Section 4 for details). Finally, a small percentage of a commercial fragrance was added to complete the formulation.

The final gel (G8) was adjusted to pH 5.5 using lactic acid (see Section 4 for full details).

**Figure 11:**
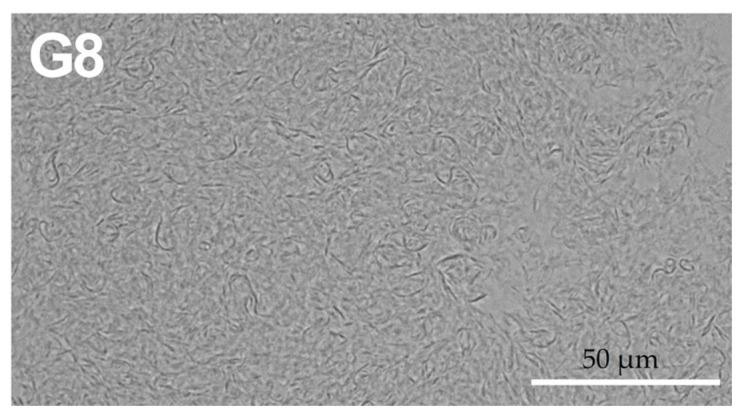
Optical microscope images of gel G8. Scalebar is 50 μm.

The addition of other components does not impair gel formation, further confirming the robustness of SCG as a gelator. Optical microscopy of the wet gel at 40× magnification (Figure 11) revealed a fibrous network similar to that observed in G1. However, the fibers in this sample are shorter and thicker, suggesting differences in the stability and structural organization of the gel network.

**Figure 12:**
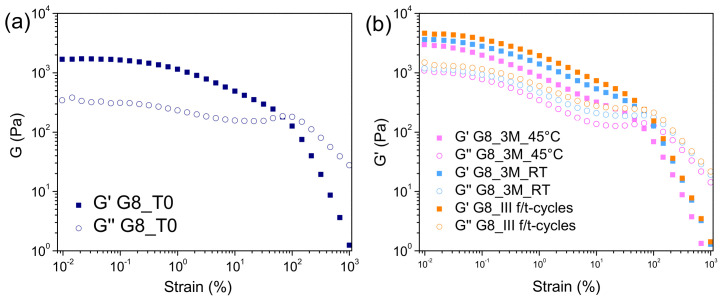
(**a**) Amplitude sweep of G8 (blue royal); (**b**) amplitude sweep of the stability tests: after 3 months at rt (pale blue), after 3 months at 45 °C (pink), after 3 freeze and thaw cycles (orange).

The properties of G8 were further investigated by rheological analysis, which confirmed its gel-like behavior, as shown by the predominance of the elastic modulus over the viscous component under the tested conditions (Figure 12 and Figure S5). G8 was also subjected to stability tests under accelerated aging conditions, including storage at room temperature for 3 months, 45 °C for 3 months, and three freeze–thaw cycles (−15 °C for 30 days followed by 1 day at room temperature, repeated three times). After these treatments, amplitude sweep tests confirmed the robustness of the system, with no significant changes in its mechanical properties (Figure 12 and Figure S6). Overall, these results demonstrate that the SCG gel network can accommodate the tested cosmetic components while maintaining its structural integrity, highlighting its suitability for practical formulation development.

Foamability, defined as the ability of a formulation to generate foam, is a key parameter in the development of new shampoos. This property is particularly important in detergent applications, where it significantly affects both product performance and consumer perception. To evaluate the foaming ability of G8 and assess its suitability as a supramolecular shampoo, a simple Bartsch test was performed. The Bartsch method [44] is a straightforward foam characterization technique in which a vial of volume V_(vial)_ containing a small amount of solution (V_1_ < V_(vial)_) is vigorously shaken by hand to generate foam. Although the results may be influenced by operator-dependent factors, such as shaking frequency and amplitude, the method provides a rapid preliminary assessment of foaming behavior.

**Figure 13:**
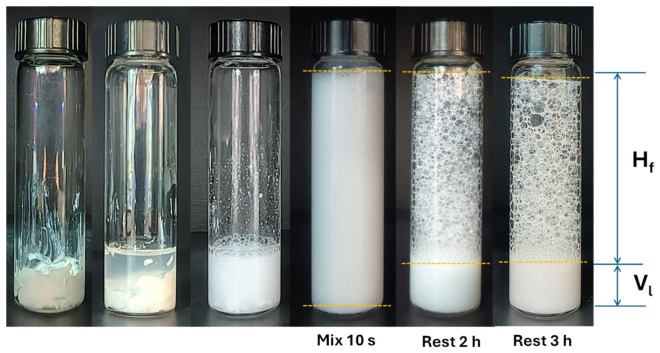
Photos of foam formed immediately after shaking the 20 mL vial with a solution of the final shampoo G8 in sink water (4 mL). The dashed lines highlight the limits of the foam, separating it from the bottom layer of liquid reformed after shaking and from the air layer on the top.

Because anionic surfactants such as SCG are sensitive to Ca^2+^ and Mg^2+^ ions, G8 was diluted (0.25 g mL^−1^) in 4 mL of tap water with a hardness of 40 °f to evaluate its performance under realistic conditions (Figure 13). The test demonstrated that foam formation is not hindered even in hard water. Moreover, despite the supramolecular nature of the formulation, in which the surfactant is self-assembled and also acts as a rheological modifier, G8 generated abundant foam that remained stable for several hours.

A closer analysis of the G′ values reported in this work and summarized in Table 1 enables a direct comparison of the mechanical strength of the different gels. Starting from the strong gel formed by pure SCG, the progressive addition of the various ingredients leads to an overall decrease in mechanical strength from 53.1 kPa of G1 to 1.7 kPa of G8. This progressive softening of the final formula is not concerning and perfectly aligns with the rheological and sensorial properties of a shampoo. Finally, the aged samples of G8 retain the same properties, showing G’ values in the range of 2–4 kPa depending on the environmental conditions.

In contrast, gels based on SCA show a much more pronounced reduction in strength—by nearly two orders of magnitude—upon the sole addition of CAPB, decreasing from 13.4 kPa to 0.2 kPa. This substantial weakening prevents the incorporation of further components. These findings are in good agreement with the pK_a_ analysis of the materials, highlighting how a thorough understanding of the physicochemical properties of the raw components is essential for the rational design of complex, application-oriented formulations with potential commercial relevance.

## 3. Conclusions

In this work, we show that amino acid-based surfactants, and in particular SCG, can act not only as mild cleansing agents but also as effective supramolecular gelators, thereby enabling the development of a fully formulated “supramolecular shampoo”. Starting from fundamental studies on self-assembly and pK_a_ behavior, we identified a correlation between molecular properties and macroscopic rheological performance, suggesting the importance of physicochemical understanding in formulation design.

Among the systems investigated, SCG exhibited superior robustness compared to SCA, maintaining strong gelation ability even upon incorporation of additional ingredients. In contrast, SCA-based systems showed a marked loss of mechanical strength, preventing further formulation development. The progressive addition of typical cosmetic components, including surfactants, preservatives, conditioning agents, and fragrance, led to the preparation of a complete supramolecular shampoo formulation, exhibiting a controlled decrease in gel strength, while preserving the essential viscoelastic and pseudoplastic behavior required for practical applications.

Importantly, the SCG-based network demonstrated excellent compatibility with all tested additives, retaining structural integrity and stability under accelerated aging and freeze–thaw conditions performed over 3 months. The final formulation (G8) exhibited satisfactory mechanical properties and long-term stability, confirming its potential as a viable prototype for real applications.

Overall, this study highlights how concepts from supramolecular chemistry can be successfully translated into complex, application-oriented systems. By leveraging reversible, non-covalent interactions, it may be possible to design sustainable and functional materials that have the potential to meet industrial requirements, offering a possible alternative to conventional polymer-based non-biodegradable thickeners in cosmetic formulations.

## 4. Materials and Methods

*Materials.* Sodium cocoyl alaninate (30% in water) (Eversoft™ ACS-30S) was purchased from Sino Lion USA (Florham Park, NJ, USA); sodium cocoyl glycinate (22% in water) (Galsoft SCG) was purchased from Galaxy Surfactants Ltd. (Navi Mumbai, India); cocamidopropyl betaine (30% in water) (Empigen^®^ BS/FA Betaine) was purchased from Innospec Performance Chemicals (Salisbury, NC, USA); decyl glucoside (53% in water) (Plantacare^®^ 2000 UP) was purchased form BASF (Ludwigshafen am Rhein, Germany); cetrimonium chloride (25% in water) (Quafin CT) was purchased from Farmalabor SRL (Canosa di Puglia, Italy); phenoxyethanol (90%) and ethylhexylglycerin (10%) (Euxyl™ PE 9010) was purchased form Ashland Global Specialty Chemicals Inc. (Covington, GA, USA); caprylhydroxamic (5%)/1,2-hexanediol (30%)/propanediol (65%) (Spectrastat PHL) was purchased from Inolex Inc. (Philadelphia, PA, USA); benzylic alcohol (99%) purchased from IMCD (Milan, Italy); sodium benzoate (99%) was purchased from A.C.E.F. (Fiorenzuola d’Arda, Italy); lactic acid FU-BP E 270 (90% in water) purchased from A.C.E.F. (Fiorenzuola d’Arda, Italy); the fragrance was purchased from GRC Parfum (Settimo Milanese, Italy).

Preparation of G1 or G2: SCA or SCG (10% *w*/*w*) + CAPB (4.5% *w*/*w*)

The anionic surfactant (SCA or SCG) was first weighed, followed by the addition of cocamidopropyl betaine (CAPB). Deionized water was then added to reach the final volume, and the mixture was left under magnetic stirring for at least 15 min. The pH was subsequently adjusted with 90% L-lactic acid. The formulation was fully prepared at r.t. (25 °C).

Preservative study—Gels G3–G6

The anionic surfactant (SCG) was weighed and combined with CAPB. Part of the deionized water was added, and the mixture was stirred using an overhead stirrer at 300 rpm for at least 15 min. The preservative, when in solid form, was pre-solubilized in the remaining portion of water before being added to the formulation. Stirring continued at 300 rpm for 15 min to obtain a homogeneous solution, and the pH was then adjusted using 90% L-lactic acid. The formulation was fully prepared at r.t. (25 °C). The quantities are reported in Table 2.

Preparation of G7

The anionic surfactant (SCG) was weighed and combined with CAPB. Part of the deionized water was added, and the mixture was stirred using an overhead stirrer at 300 rpm for at least 15 min. The two preservatives selected were pre-solubilized in the remaining portion of water before being added to the formulation. Stirring continued at 300 rpm for 15 min to obtain a homogeneous solution, and the pH was then adjusted using 90% L-lactic acid. The formulation was fully prepared at r.t. (25 °C). The quantities are reported in Table 3.

Preparation of G8

A total batch of 600 g was prepared according to the following procedure.

In the first beaker, SCG was mixed with CAPB under agitation using an overhead stirrer at 300 rpm.

In a second beaker, the fragrance, benzyl alcohol, sodium benzoate and Decyl Glucoside were mixed together until complete homogenization.

The solution in the first beaker was combined with the second, under agitation using an overhead stirrer set at 300 rpm. The conditioning agent cetrimonium chloride was directly added to the formula.

In the meantime, the trigger solution was prepared separately by dissolving lactic acid in the remaining amount of deionized water. Finally, the trigger solution was added slowly to the system under agitation, and the mixture was left under stirring (300 rpm) until a homogeneous formulation was obtained. The formulation was fully prepared at r.t. (25 °C). The quantities are reported in Table 4.

Methodology for the determination of the apparent pKa.—A XS pH70 Vio Portable pHmeter (XS Instruments, Carpi (MO), Italy) with a 2-pore steel T electrode was employed for all pH measurements. The stated accuracy of the pH measurements is ±0.1. The pH meter was calibrated before each experiment to check the response of the electrode. Depending on the desired concentration, considering a total volume of 2.5 mL for each sample, the required amount of gelator was suspended in Milli-Q^®^ H_2_O and the pH was increased to 10.2 by adding NaOH 1M. The samples were stirred to fully dissolve the gelator. The pKa values of gelator solutions were determined by titration via the addition of aliquots of a 1.0 M HCl (VWR Chemical, Milan, Italy) solution in triplicate. pH values were recorded until they reached a stable value after each addition during the titration process. Due to the strong and constant agitation applied, samples were always liquid during the titration experiments.

Rheological analysis—The rheological analyses were performed using an Anton Paar (Graz, Austria) MCR 92 rheometer. Rotational control shear rate analyses were performed at 23 °C collecting 37 data points at shear rate (%) between 0.001 and 1000 to evaluate the viscosity profile using a cone (d = 50 mm)/plane measuring system was used, with a gap of 0.098 mm. Oscillatory amplitude sweep experiments were performed at 25 °C using a Peltier control system and plate/plate geometry (shaft PP25/P2). Data points were collected (γ: 0.01–100%) using a constant angular frequency of 1 Hz. Temperature Sweeps were performed to predict products stability toward temperature cycles. The tests were carried out using the shaft PP25/P2 and were performed between 23 and 55 °C collecting 64 data points, one every 0.51 min. During the analysis, the frequency of 1 Hz was kept constant as well as the shear strain that was selected, from time to time, in the linear viscoelastic range of the sample. The analysis was then repeated on the same sample from 55 to 23 °C.

Suspending properties—To evaluate the suspending properties, beads made of jojoba esters Florabeads^®^ Jojoba 40/60 purchased from Crgill Beauty (Chandler, Arizona) INCI: Jojoba Esters, having a density < 1 g/cm^3^, together with silica beads Silicami™ 200/500 purchased from Croda International (Snaith, UK) INCI: Hydrated Silica, having a density >1 g/cm^3^ were used. Each of them was inserted in the formulation at 1% (*w*/*v*). For the white formulation, 0.075 g of colorant solution (Color Index 14700 at 2.5% *w*/*v*) was added to 20 g of formulation to make the silica particles visible. The suspending properties were evaluated in 15 mL glass squat vials at both 45 °C and R.T. for three months.

Foamability—The Bartsch method [44] was adopted to generate the foam. A cylindrical vial of volume: V_vial_ = 20 mL (internal diameter, D ≈ 18 mm), and height, h ≈ 83 mm was used. The final shampoo (G8) was weighed in the vial (1 g), and 4 mL of tap water V_l_ (hardness 40 °f) were poured in the vial using a pipette. After a gentle mixing to help the dissolution while avoiding foaming, the vial was sealed using a cap. The foam was generated by manually shaking the vial vigorously for 10 s.

The experiment was repeated twice to ensure the repeatability of the measurement, always at r.t. (25 °C).

Stability—Stability studies were conducted using a thermostatic stove Memmert (Schwabach, Germany) set at 45 °C and −15 °C. The pH of the samples was measured with a pH meter XS pH 8 PRO Basic equipped with an XS Sensor Standard T BNC.

## Figures and Tables

**Table 1:** Comparison of the properties of gels from SCG and SCA in different conditions. In any case, the pH is adjusted with lactic acid to 5.5. G’ values taken at γ = 0.02%.

Gel	Components	G′ (KPa)
Ref. [22]	SCG/LA	53.1
Ref. [22]	SCA/LA	13.4
G1	SCG/LA/CAPB	14.3
G2	SCA/LA/CAPB	0.2
G3	SCG/LA/CAPB/BnOH	6.9
G4	SCG/LA/CAPB/PhCOOH	2.8
G5	SCG/LA/CAPB/caprylhydroxamic acid/1,2-hexanediol/1,2-propanediol	0.7
G6	SCG/LA/CAPB/phenoxyethanol/ethylhexylglycerin	3.5
G7	SCG/LA/CAPB/BnOH/PhCOOH	4.3
G8	SCG/LA/CAPB/BnOH/PhCOOH/decyl glucoside/fragrance/cetrimonium chloride	1.7
G8 (3m at RT)	G8 after 3 months at rt	3.5
G8 (3m at 45 °C)	G8 after 3 months at 45 °C	2.8
G8 (3 cycles)	G8 after 3 freeze/ thaw cycles	4.5

**Table 2:** Composition of **G3–G6** prepared with different preservatives and their respective concentrations.

INGREDIENTS	G3	G4	G5	G6
	(% *w*/*w*)	(% *w*/*w*)	(% *w*/*w*)	(% *w*/*w*)
SCG (commercial solution at 22%)	45.40	45.40	45.40	45.40
CAPB (commercial solution at 30%)	15.00	15.00	15.00	15.00
lactic acid	2.40	2.40	2.40	2.40
benzylic alcohol	0.50	/	/	/
sodium benzoate	/	0.19	/	/
caprylhydroxamic acid/1,2-hexanediol/propanediol	/	/	1.50	/
phenoxyethanol and ethylhexylglycerin	/	/	/	0.90
deionized water	36.70	37.00	35.70	36.30

**Table 3:** Composition of G7, including the selected preservatives.

INGREDIENTS	G7
	(% *w*/*w*)
SCG (commercial solution at 22%)	45.40
CAPB (commercial solution at 30%)	15.00
benzylic alcohol	0.50
sodium benzoate	0.19
lactic acid	2.40
deionized water	36.51

**Table 4:** Composition of G8.

INGREDIENTS	FUNCTION	% *w*/*w*
SCG	Rheological modifier and anionic surfactant	45.50
CAPB	Zwitterionic surfactant	15.00
lactic acid	pH modifier and trigger	2.30
benzyl alcohol	Preservatives	0.50
sodium benzoate		0.19
decyl glucoside	Non-ionic surfactant	2.50
cetrimonium chloride	Cationic surfactant, conditioning agent	2.00
fragrance	Perfume	0.60
water to 100	Solvent	31.41
TOT	-	100.00

## Data Availability

The data underlying this study are available in the published article and its online Supporting Information. Raw data comprising the pKa of the compounds reported and the rheological studies on the gels are openly available in AMSActa Institutional Research Repository DOI: http://doi.org/10.6092/unibo/amsacta/9039.

## Supplementary Materials

The following supporting information can be downloaded at: https://www.mdpi.com/article/10.3390/gels12070589/s1. Table S1: Commercial surfactants used with their relative percentage of active matter; Figure S1: Viscosity curves and frequency sweep of gels G1 and G2; Figure S2: Viscosity curves of formulations G3, G4, G5, and G6; Figure S3: Frequency sweep of formulations G3, G4, G5, and G6; Figure S4: Viscosity curves and frequency sweep of formulations G7; Figure S5: Viscosity curves and frequency sweep of G8 at T0; Figure S6: Viscosity curves of the accelerated stability tests of G8.

## Abbreviations

The following abbreviations are used in this manuscript:

SLES	sodium laureth sulfate
CAPB	cocamidopropyl betaine
SCG	sodium cocoyl glycinate
SCA	sodium cocoyl alaninate
