# Supplementary material for: From Fundamental Self-Assembly Studies to Applications in Everyday Life: The Formation of a Supramolecular Shampoo
Source: Gels. 2026 Jul 2;12(7):589. doi: 10.3390/gels12070589 (PMC13409622; doi:10.3390/gels12070589)
Supplement: Supplementary file 1 [file gels-12-00589-s001.zip › gels-4387093-supplementary.pdf]

SUPPORTING INFORMATION

From Fundamental Self-Assembly Studies to Applications in Everyday Life: the Formation of a Supramolecular Shampoo

Sofia Chinelli [1], Roberta Stile [1], Demetra Giuri [1,*] and Claudia Tomasini [1,*]

[1] Dipartimento di Chimica Giacomo Ciamician - Università di Bologna - Via Piero Gobetti, 85 – 40129 Bologna – Italy.

Table S1. Commercial surfactants used with their relative percentage of active matter. Page S2

Figure S1. Viscosity curves and frequency sweep of gels **G1** and **G2**. Page S3

Figure S2. Viscosity curves of formulations **G3**, **G4**, **G5**, and **G6**. Page S4

Figure S3. Frequency sweep of formulations **G3**, **G4**, **G5**, and **G6**. Page S5

Figure S4. Viscosity curves and frequency sweep of formulations **G7**. Page S6

Figure S5. Viscosity curves and frequency sweep of **G8** at T0. Page S6

Figure S6. Viscosity curves of the accelerated stability tests of **G8**. Page S7

Table S1. Commercial surfactants used with their relative percentage of active matter.

SURFACTANTS	**AVERAGE % OF ACTIVE MATTER IN THE COMMERCIAL RAW MATERIAL**
**SCA**	30%
**SCG**	22%
**CAPB**	30%

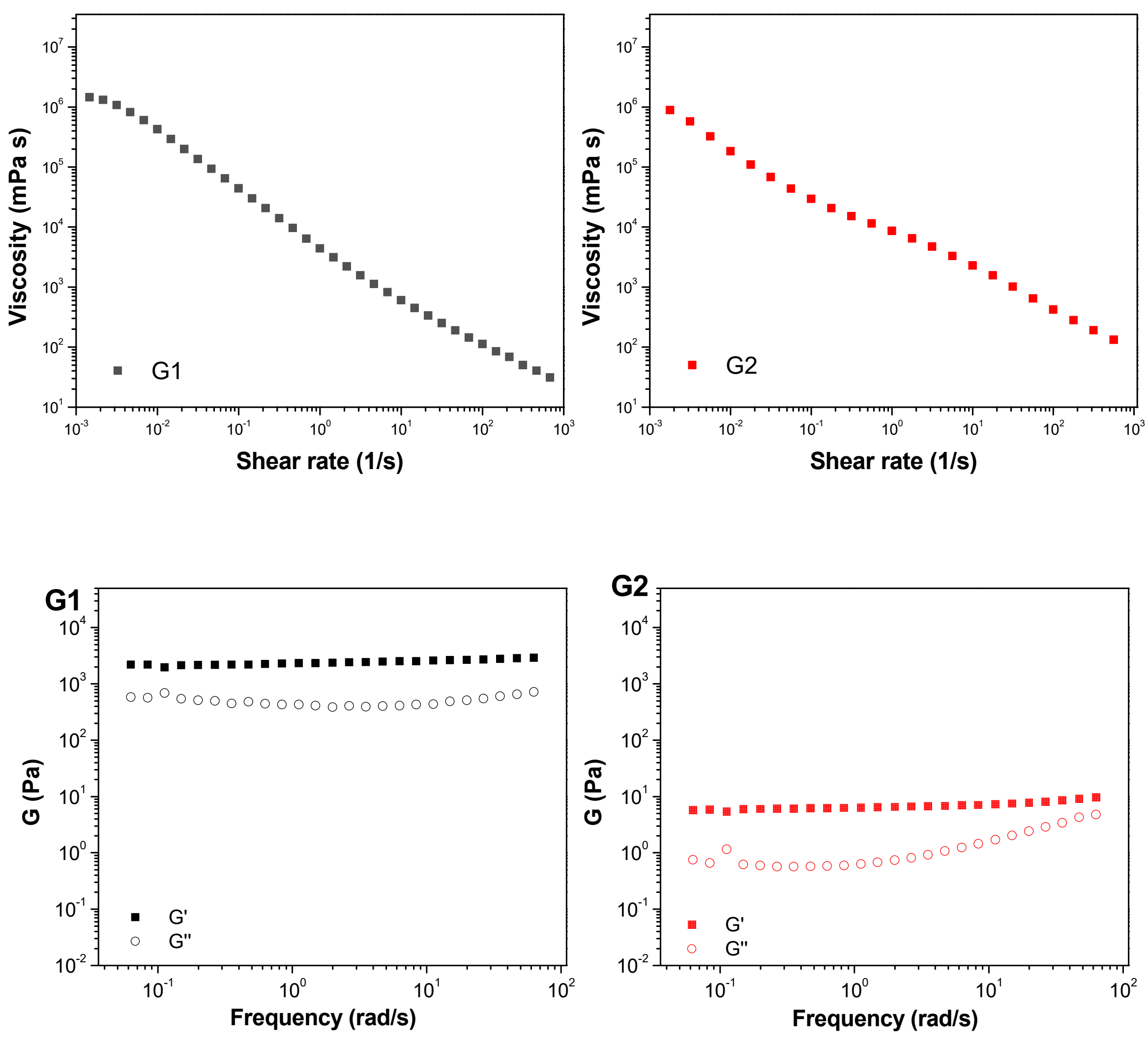

Figure S1. *Top*. Viscosity curves of **G1** (left) and **G2** (right). *Bottom.* Frequency sweep of **G1** (left) and **G2** (right).

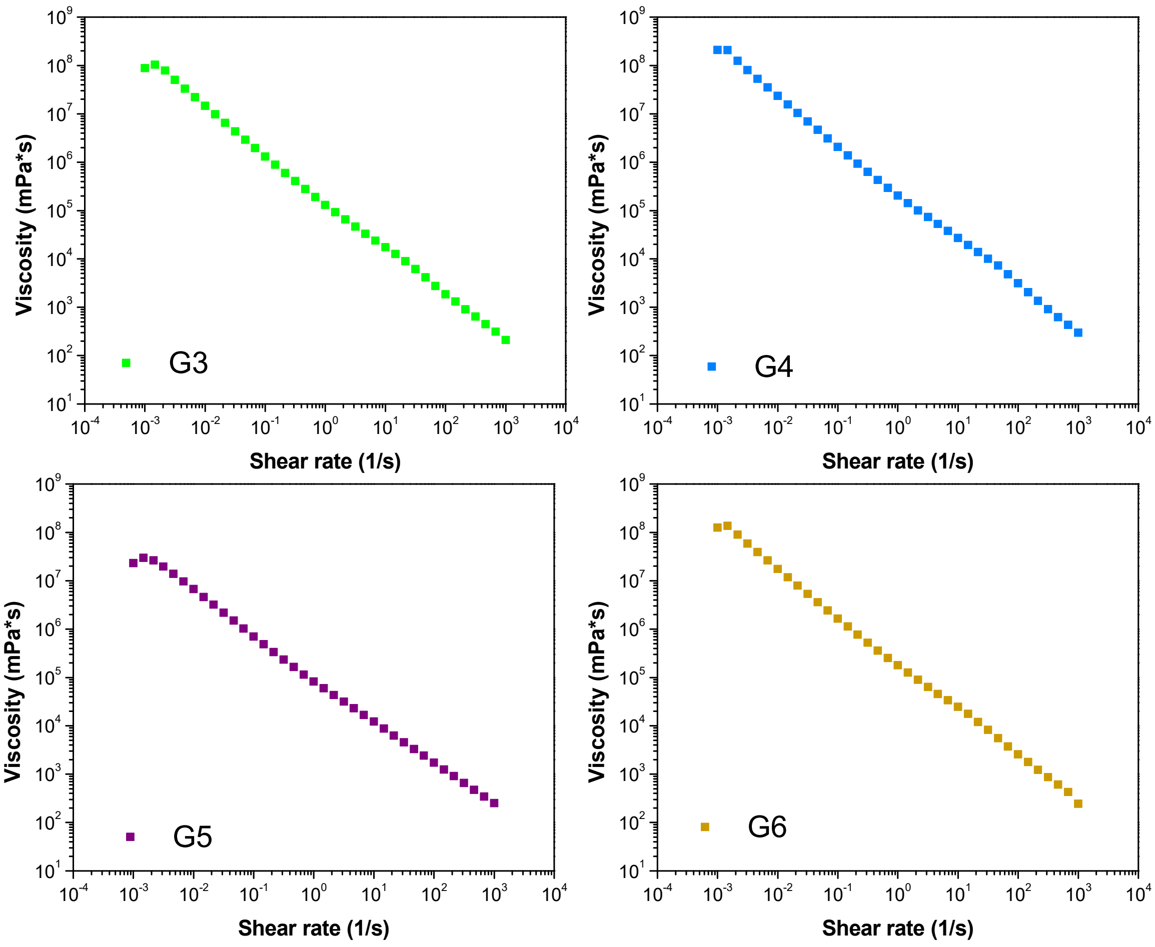

Figure S2. Viscosity curves of **G3**, **G4**, **G5** and **G6**.

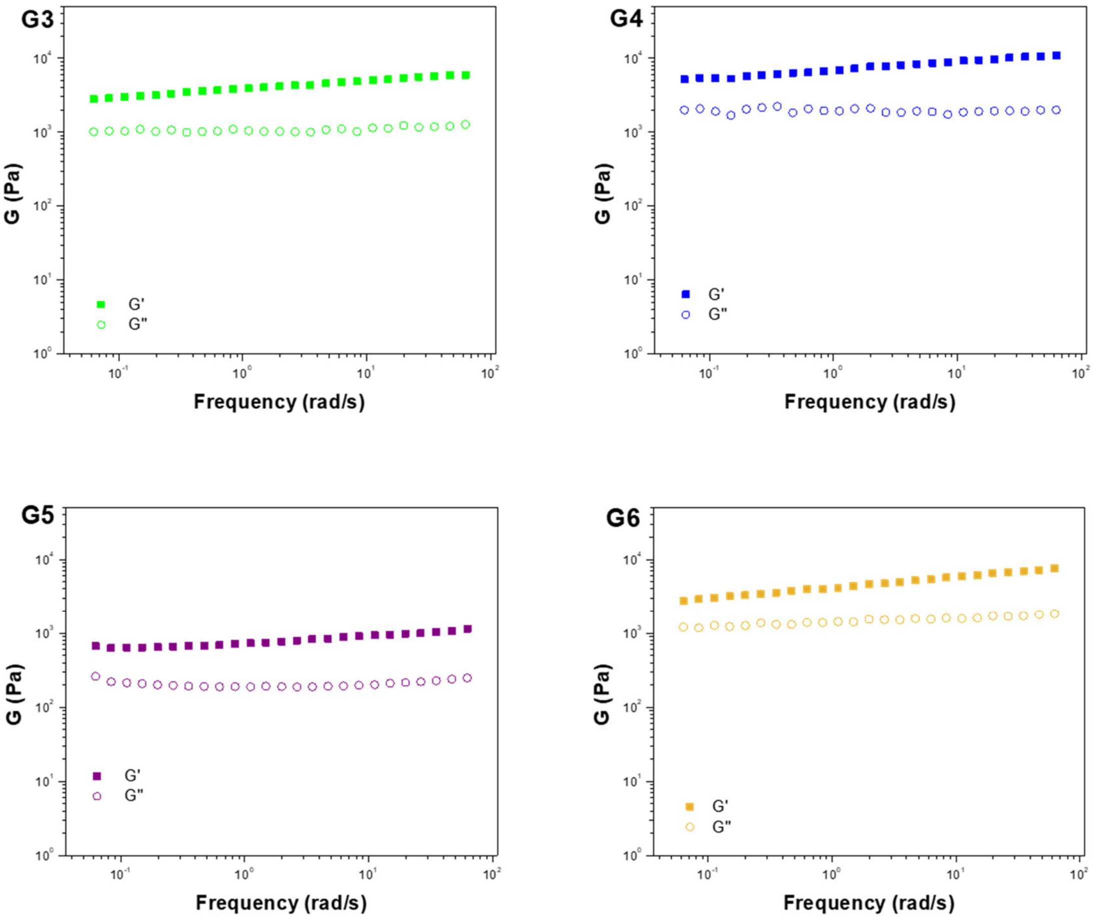

Figure S3. Frequency sweep of **G3**, **G4**, **G5** and **G6**.

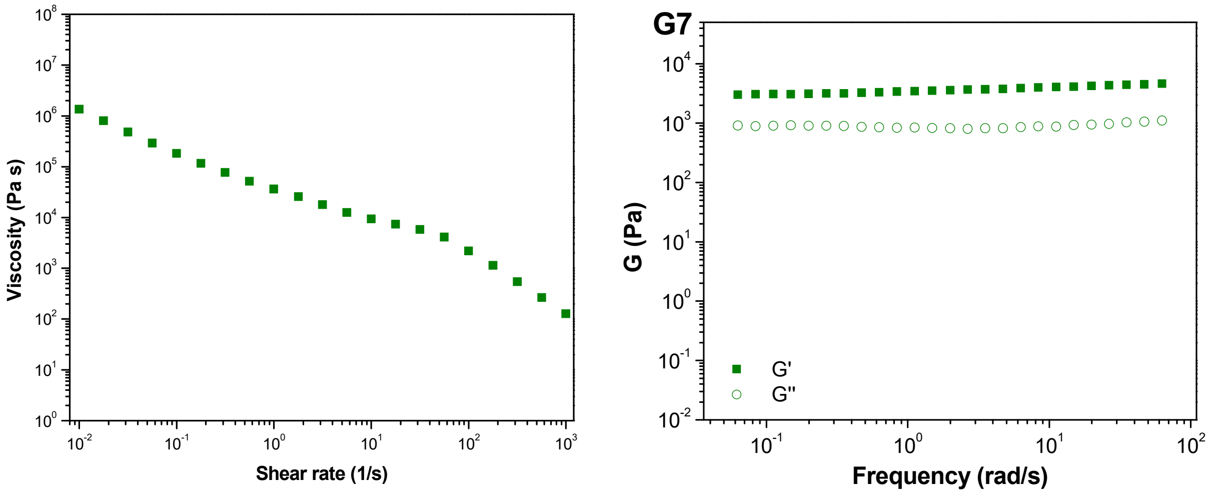

Figure S4. Viscosity curve (left) and frequency sweep (right) of **G7** including the two preservatives selected.

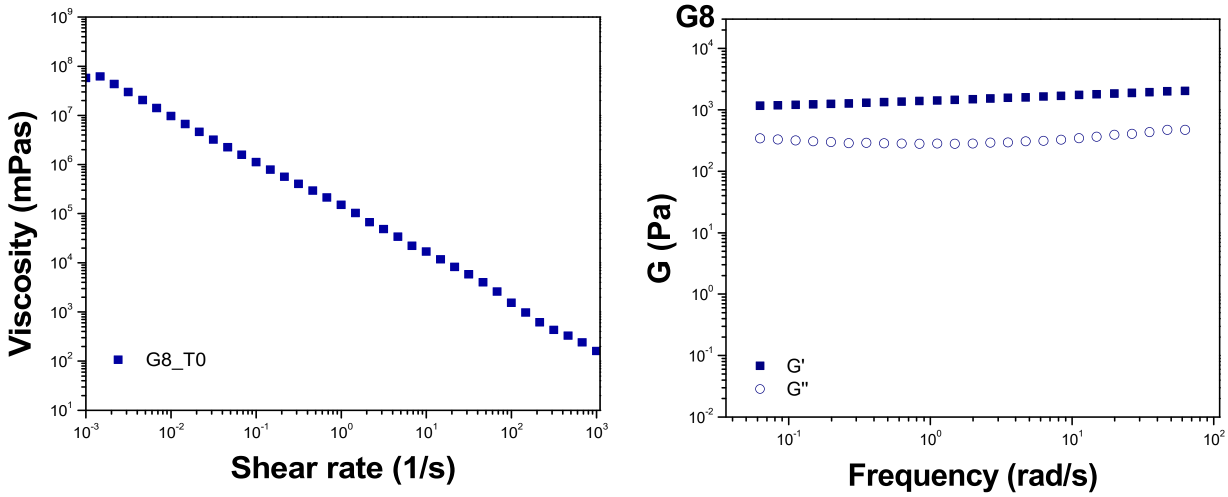

Figure S5. Viscosity curve and frequency sweep (right) of **G8** (left) at T0.

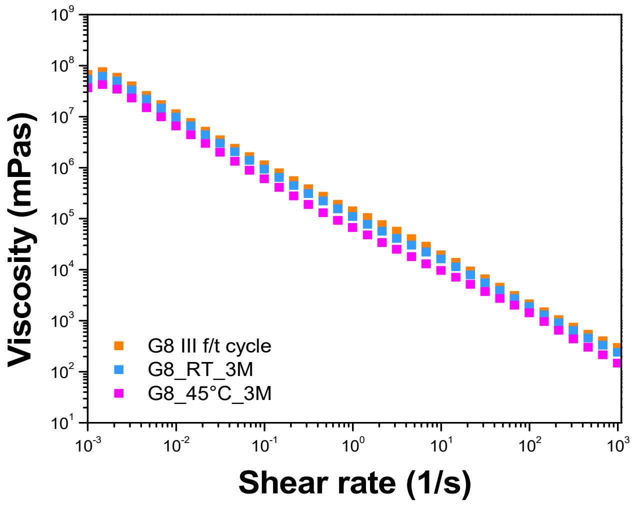

Figure S6. Viscosity curve of the accelerated stability test of **G8**: after 3 months at RT (pale blue), after 3 months at 45 °C (pink), after 3 freeze and thaw cycles (orange).
